# Initial Surgical Experience with Aortic Valve Repair: Clinical and Echocardiographic Results

**DOI:** 10.5935/1678-9741.20160027

**Published:** 2016

**Authors:** Francisco Diniz Affonso da Costa, Daniele de Fátima Fornazari Colatusso, Ana Claudia Brenner Affonso da Costa, Eduardo Mendel Balbi Filho, Vinicius Nesi Cavicchioli, Sergio Augusto Veiga Lopes, Andrea Dumsch de Aragon Ferreira, Claudinei Collatusso

**Affiliations:** 1 Pontifícia Universidade Católica do Paraná (PUCPR), Curitiba, PR, Brazil; 2 Department of Cardiothoracic Surgery do Mount Sinai Hospital, New York, United States; 3 Instituto de Neurologia e Cardiologia de Curitiba (INC), Curitiba, PR, Brazil; 4 Hospital Irmandade Santa Casa de Curitiba, Curitiba, PR, Brazil

**Keywords:** Heart Valve Diseases, Aortic Diseases, Aortic Valve Insufficiency, Aortic Valve

## Abstract

**Introduction:**

Due to late complications associated with the use of conventional prosthetic heart valves, several centers have advocated aortic valve repair and/or valve sparing aortic root replacement for patients with aortic valve insufficiency, in order to enhance late survival and minimize adverse postoperative events.

**Methods:**

From March/2012 thru March 2015, 37 patients consecutively underwent conservative operations of the aortic valve and/or aortic root. Mean age was 48±16 years and 81% were males. The aortic valve was bicuspid in 54% and tricuspid in the remaining. All were operated with the aid of intraoperative transesophageal echocardiography. Surgical techniques consisted of replacing the aortic root with a Dacron graft whenever it was dilated or aneurysmatic, using either the remodeling or the reimplantation technique, besides correcting leaflet prolapse when present. Patients were sequentially evaluated with clinical and echocardiographic studies and mean follow-up time was 16±5 months.

**Results:**

Thirty-day mortality was 2.7%. In addition there were two late deaths, with late survival being 85% (CI 95% - 68%-95%) at two years. Two patients were reoperated due to primary structural valve failure. Freedom from reoperation or from primary structural valve failure was 90% (CI 95% - 66%-97%) and 91% (CI 95% - 69%-97%) at 2 years, respectively. During clinical follow-up up to 3 years, there were no cases of thromboembolism, hemorrhage or endocarditis.

**Conclusions:**

Although this represents an initial series, these data demonstrates that aortic valve repair and/or valve sparing aortic root surgery can be performed with satisfactory immediate and short-term results.

**Table:** 

Abbreviations, acronyms & symbols	
AAo	= Aortic root or ascending aorta
AI	= Aortic valve insufficiency
AVR	= Aortic valve replacement
CPB	= Cardiopulmonary bypass
ELH	= Effective leaflet height
FML	= Leaflet free margins length
LGH	= Leaflet geometric height
STJ	= Sino-tubular junction
SV	= Sinuses of Valsalva
SVD	= Structural prosthetic valve dysfunction
TEE	= Transesophageal
TTE	= Transthoracic echocardiography

## INTRODUCTION

Conventional surgical treatment for aortic valve insufficiency (AI), with or without aortic root or ascending aorta (AAo) dilatation, consists of isolated aortic valve replacement (AVR) or total root replacement with the Bentall operation^[1]^. Although immediate surgical results are excellent, patients are exposed to the cumulative risks of late prosthetic heart valve complications, including thromboembolic events, structural (SVD) and nonstructural prosthetic valve dysfunction, bacterial endocarditis and reoperations, not to say the inconvenient of permanent anticoagulation in the case of mechanical substitutes^[1-3]^. Frequently these procedures are performed in young patients, which is associated with excess mortality when compared to the normal age and gender matched population^[4]^.

In theory, AVR and/or aortic valve sparing operations can restore near normal function and hemodynamics of the aortic valve, reducing or eliminating complications associated with conventional prosthetic heart prosthesis^[5]^. On the other hand, aortic valve repair is associated with a steep learning curve and requires a detailed knowledge and understanding of the anatomy and functional inter-relation of all involved structures, including the aortic cusps, annulus, sinuses of Valsalva (SV), sinotubular junction (STJ) and AAo^[6-8]^.

Although the theoretical principles for the conservative management of the aortic valve are well established^[9]^, several different surgical techniques have been described due to the wide anatomical variations presented during the operations^[10,11]^. However, the long-term results available does not permit to demonstrate the superiority of one specific technique, making aortic repair still subjective, individually based and depending on the best surgeon judgment^[12]^. More recently, Lansac et al.^[6]^ have proposed a systematic surgical approach based on well-defined anatomical criteria.

Even taking into account these limitations, several groups have reported excellent immediate results and very encouraging durability with up to 15-20 years of follow-up^[5,10]^. Our experience with aortic valve repair began in 2012, and seemed appropriate to review our immediate and short-term clinical and echocardiographic outcomes, in order to compare with those reported in the international literature and define whether the application of these techniques is safe and reproducible in our midst.

## METHODS

This study was submitted to the Research Ethics Committee of Instituto de Neurologia e Cardiologia de Curitiba and is registered under number 42707915.4.0000.5227 in Brazil Platform.

From March 2012 to March 2015, 37 patients with AI ± aortic root dilatation consecutively underwent aortic valve repair at Institute of Neurology and Cardiology of Curitiba (INC Cardio) and Santa Casa de Curitiba PUCPR. The average age was 48±16 years (min=21, max=79) and 30 (81%) were male. The aortic valve was bicuspid in 20 (54%) patients and tricuspid in 17 (46%). Nineteen (51%) patients had significant dilation of the aortic root defined as a diameter greater than 45 mm. Demographic data are summarized in Table 1.

**Table 1:** Demographic data and clinical profile.

Variables	n (%)
Gender	
Male	30 (81%)
Female	7 (19%)
Age (years)	
21-40	15 (40%)
41-60	10 (27%)
>61	12 (33%)
Race	
White	31 (84%)
Mestice	2 (5%)
Unknown	4 (11%)
Etiology	
Bicuspid	17 (46%)
Rheumatic	1 (3%)
Aortic root dilatation*	19 (51%)
Acute aortic dissection	1 (3%)
Aortitis	1 (3%)
Endocarditis	2 (5%)
Associated conditions	
Ascending aorta aneurysm	22 (54%)
Subaortic stenosis	1 (3%)
Mitral insufficiency	4 (10%)
Tricuspid insufficiency	1 (3%)
Coronary artery disease	1 (3%)
Aortic insufficiency	
Mild	1 (3%)
Moderate	12 (32%)
Severe	24 (65%)
LV systolic dimension	41±7.5 (30 - 61)
LV diastolic dimension	62±7.7 (47 - 80)
Ejection fraction	60±7.4 (45 - 72)
NYHA functional class	
I	10 (27%)
II	6 (16%)
III	5 (14%)
IV	7 (19%)
Unknown	9 (24%)
Comorbidities	
Diabetes	2 (6%)
SAH	19 (51%)

* Some patients with aortic root dilatation had bicuspid aortic valves.

LV=left ventricle; NYHA=New York Heart Association; SAH=systemic arterial hypertension

Preoperative evaluation included, according to the specific need in each case individually, transthoracic echocardiography (TTE) and/or transesophageal (TEE), chest computed tomography scan or coronary angiography in order to obtain the specific anatomical details involved in valvular dysfunction. Whenever anticipated the possibility of performing an aortic valve repair, all available surgical alternatives were discussed with each patient. For those who opted for a valve repair strategy, the other options were already chosen, in case a conservative approach was not feasible at the time of the operation.

### Surgical Technique

In all cases, monitoring was performed with intraoperative TEE used to quantify and analyze the mechanisms involved in valvular insufficiency preoperatively and to ascertain adequate post-surgical valve competence.

The operations were performed with a median thoracotomy with cardiopulmonary bypass (CPB) and moderate hypothermia at 32°C. In cases where an open distal anastomosis was required, the systemic temperature was reduced to 25°C for adequate cerebral protection. Myocardial protection was achieved with infusion of intermittent cold blood cardioplegic solution directly into the coronary ostia. The mean aortic clamping was 85±23 min (min=45, max=131) and the CPB was 108±28 min (min=60, max=170).

Routinely, a wide circumferential dissection of the aortic root was performed to a level below the lowest point of the cusps insertion in the aortic annulus. This can be laborious and tricky, especially in the area of the right coronary cusp attachment, but right ventricular outflow and septal muscle should be thoroughly dissected away from the aortic wall at this region. After aortic cross-clamping, ascending aorta was circumferentially transected approximately 1 cm above the STJ, and proper exposure of the native valve was obtained. Assessment of aortic valve geometry is not easy in the empty and arrested heart. By using 3 commissural traction sutures, the axis of the aortic valve may be oriented towards the surgeon's view. Moreover, by applying a vertical and outward tension on those sutures, the cusps can be stretched, facilitating the comparison between the free margin's height and length.

Valve analysis included meticulous observation of every anatomical structure, including the dimensions of the aortic annulus, the sinuses and STJ, and detailed inspection of the valve cusps, paying attention to its texture and the presence of fenestrations. The judicious judgment of the dimensions of the leaflets included the evaluation of its geometric height (LGH), the length of their free margins (FML) and its insertion in the aortic annulus to estimate the possible degree of prolapse in one or more cusps.

Prolapse identification was performed by comparing the height of one leaflet with the adjacent one, and/or by measuring its effective height (ELH) with the aid of a Schaefers caliper. In the case of prolapse of 1 or 2 cusps, the reference height can be taken from the nonprolapsing cusp or cusps. Whenever identified some degree of prolapse, the same has been corrected by reducing the length of the free edge through central plication with 6-0 polypropylene sutures. When this plication was quite extensive, the excess resulting tissue in the middle of the leaflet was resected and reapproximated with interrupted sutures. Prolapse of all 3 cusps is rare in the native AV, but it occasionally can be induced after a valve-sparing procedure. In this situation, the reference used to correct prolapse can be the middle height of the commissures, or it can be determined with the Schaefer's caliper. In type 0 bicuspid valves, cusp prolapse correction is performed as for tricuspid AVs, with either a nonprolapsing cusp as the reference or restoration of the height of coaptation to the middle height of the commissures. In type 1 valves, the median raphe is addressed first. If the raphe is relatively mobile and only mildly thickened and fibrosed, it is preserved and shaved. If the raphe is restrictive or calcified, a parsimonious triangular resection of this tissue is performed. Next, the quantity of remaining cusp tissue is assessed by putting the 2 arms of a 6-0 polypropylene suture on the free margin of the conjoint cusp, on either side of the resected raphe. At this point, lack of cusp restriction and good valve opening are signs of the presence of adequate cusp tissue. The leaflet edges are reapproximated primarily when adequate cusp tissue is present; in the absence of adequate tissue, a triangular autologous treated or bovine pericardial patch is used for cusp restoration. Next, the free margins of both cusps are compared for the presence of any prolapse, which is corrected with free margin plication or resuspension.

In cases with marked dilation of the aortic root (> 45 mm) , resection of all aneurysmal tissues of the SV, the STJ and AAo was performed, and the reconstruction made by the reimplantation technique described by David & Feindel^[13]^ or the remodeling technique described by Sarsam & Yacoub^[14]^. In general, we have used the David reimplantation technique for patients with dilated annulus and reserved the Yacoub technique when the diameter of the aortic annulus is the normal range. However, in some instances, annular reduction with an external circumferential Dacron strip followed by an aortic remodeling graft as proposed by Lansac et al.^[6]^ has been performed.

For the reimplantation technique, the proximal suture line is made with 6 pledgeted sutures (3 at each interleaflet triangle and 3 below the nadir of each leaflet attachment) placed from the inside the left ventricular outflow tract, and exiting outside the aortic root and geometrically anchored at proper points in the Dacron graft. With this proximal suture line, any annular dilatation will be corrected and set to the desired diameter. Then, under tension, the commissures are resuspended inside the graft and the remnants of the aortic sinus wall are carefully sutured in a scalloped fashion with running 5-0 polypropylene sutures. At this stage, cusp heights are reassessed, as induced cusp prolapse may occur, and one or two additional stitches at the free margin may occasionally be necessary. The coronary ostia are then reimplanted in their corresponding locations with running sutures of 5-0 or 6-0 polypropylene sutures. At this stage, valve competency is visually analyzed and can be estimated by pressurizing the aortic graft with cardioplegic solution. The final step is the distal anastomosis in the ascending aorta or the proximal aortic arch.

For the remodeling technique, the Dacron graft is trimmed proximally to create two or three tongues that will be sutured to the remnants of the aortic wall with running 5-0 polypropylene sutures. These sutures start at the base of the sinus wall running each side to the top of the commissures, taking care not to damage the valve leaflets. The coronary reimplantation and distal anastomosis are performed in the same fashion as in the David's technique. For the remodeling technique, the aortic annulus was reduced with an external Dacron strip whenever its diameter was greater than 25 mm.

The diameter of the Dacron graft varies depending on the technique used for aortic reconstruction, but was mainly determined based on visual analysis of the valve apparatus, by estimating the sum of FML and/or by measuring the distance between the base of subcommissural triangle between the non- and left coronary cusps up to the top of this commissure on the aortic wall. In general larger grafts are used for the reimplantation technique, as smaller grafts may over correct the aortic annulus.

**Fig. 1:**
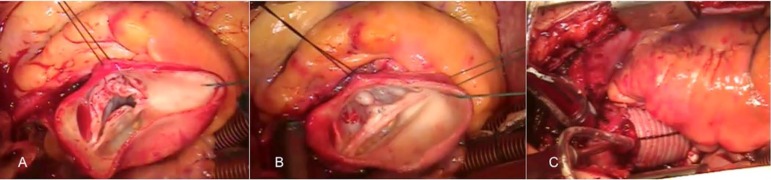
A- bicuspid aortic valve type 1 with the raphe between the left and right coronary cusp. Fibrotic thickening of the free margins and the median raphe can be seen. Severe prolapse of the fused cusp. Aorta above the STJ with important aneurysm. B - Surgical correction included the circumferential circular external reduction of the aortic annulus ring with Teflon strip, shaving of the fibrotic portions of the fused cusp and prolapse correction. At the end, both cusps are at the same level and with appropriate effective heights. C - Final aspect of the operation. The ascending aorta was replaced by Dacron tube.

**Fig. 2:**
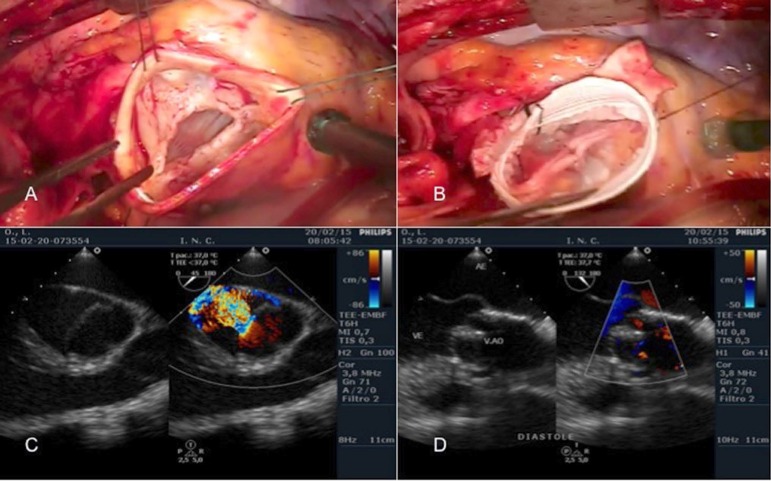
A - Bicuspid aortic valve, with thickening of the free edges, prolapse of the fused cusp. There were significant dilation of the ring and the entire aortic root. B - Correction was made by the reimplantation technique (David), and leaflet prolapse correction, requiring plication of both cusps for adequate valve competence. C - Intraoperative echocardiogram before correction, demonstrating valve prolapse and important degree of AI. D - Intraoperative echocardiogram after correction, showing good surface of coaptation of the cusps and a competent valve.

The procedures performed in our patients are listed in Table 2 and illustrated in Figures 1 and 2.

**Table 2:** Surgical data.

Surgical procedures	n (%)
Root replacement with Dacron graft	
Reimplantation (David technique)	8 (22%)
Remodeling (Yacoub technique)	11 (30%)
Plication of one or more cusps	22 (59%)
Raphe resection	2 (5%)
Free margin shaving	6 (16%)
Annular reduction - trigone to trigone	6 (16%)
Circumferential annular reduction	8 (22%)
Patch leaflet reconstruction	1 (3%)
Subcommissural plication	1(3%)
Ascending aorta replacement	22 (54%)
Associated Procedures	
Subaortic membrane resection	1 (3%)
Mitral valve repair	4 (10%)
Tricuspid valve repair	1 (3%)
Coronary artery bypass grafting	1 (3%)
Left atrial ablation	1 (3%)

### Postoperative Clinical Evaluation

All patients underwent control TTE before hospital discharge, and were asked to return in our clinic at 3, 6 and 12 months postoperatively, and yearly thereafter. In those who did not return, the information was obtained with the reference cardiologist or by direct telephone contact with patients. Clinical follow-up could be performed in all patients. The mean followup was 16±5 months (min=0.1, max=36).

Anticoagulation was indicated only in patients with associated mitral valve disease and/or those with atrial fibrillation, or in the case of a thromboembolic event postoperatively. The occurrence of postoperative complications were defined and reported in accordance with well established guidelines. For the functional analysis of the repaired valves, primary structural dysfunction was considered in any case with moderate or severe AR or a mean gradient greater than 40 mmHg.

### Statistical Analysis

Statistical analysis was performed using the "Prism 6" Mac program. Continuous variables were reported as mean ± standard deviation and were compared with Student t-test when appropriate. Categorical variables were expressed as percentages and analyzed with the chi -square test. The curves of late survival and event-free survival were built with the Kaplan-Meier method.

## RESULTS

### Early and Late Mortality

The 30-day mortality was 2.7% (1/37). This single death, due to uncontrollable intraoperative bleeding, occurred in a patient with AI and late aneurysmal dilatation of the aortic root after previous correction of an acute aortic dissection performed in another service a few months before.

The incidence of immediate postoperative morbidity was low, and included four cases of postoperative AF, a case of kidney failure not requiring dialysis and one case of stroke that caused bilateral hemianopsia with partial recovery leaving no other sequelae. Two patients required drainage puncture for presenting pericardial effusion in the first 30 days of evolution. There were no reoperations due to bleeding. The length of stay in the intensive care unit varied from 1 to 5 days, with an average of 2.1 days.

**Fig. 3:**
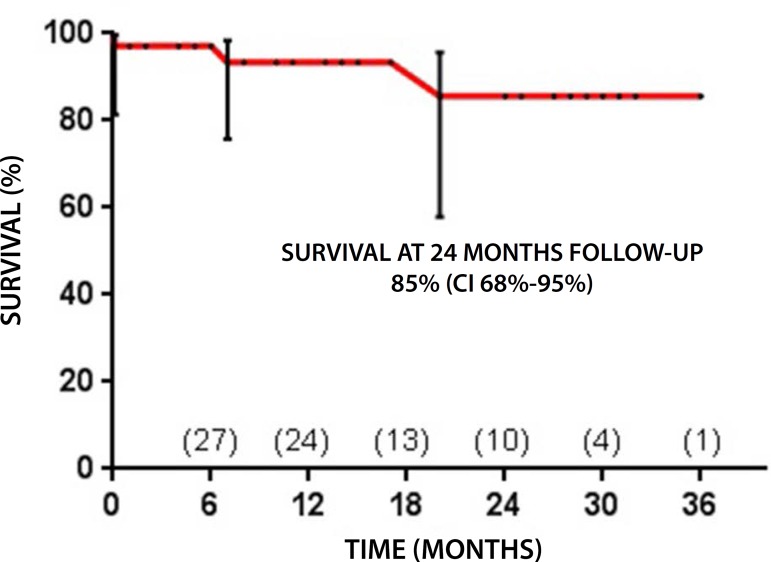
Late survival.

There were two late deaths. The first was during a reoperation for primary structural dysfunction of the valve repair, six months after the initial operation. Another patient died of cancer 20 months later. By the Kaplan-Meier estimate, late survival was 85% (CI 95% - 68%-95%) at 2 years of follow-up (Figure 3).

### Clinical Follow-up, Thromboembolism, Hemorrhage and Endocarditis

In the follow-up period, most patients showed excellent functional capacity, with 29 in functional class I, four in functional class II and only two patients, both requiring reoperation, were in class III. In the late period there was no case with thromboembolic, hemorrhagic or infectious complications.

### Reoperations

Two patients had progressive severe AI and required reoperation. The first patient had originally tricuspid valve with severe AI associated with aneurysm of the aortic root. Surgical repair consisted of aortic root replacement with a Dacron tube by the Yacoub technique plus central plication of non-coronary cusp. Although aortic valve regurgitation was only trivial in the immediate postoperative period, he developed acute AI at six months of follow-up. During reoperation, we found rupture of the coronary leaflet in the commissural region due to abrasion of the same along the suture line in the Dacron graft. He underwent an aortic homograft root replacement, but died during this procedure. The second patient with bicuspid aortic valve underwent valve repair which consisted of external reduction of the aortic annulus with a Teflon strip extending from the trigone to trigone plus extensive plication and resection of the free margin of the fused cusp. This patient developed progressive AI, requiring reoperation at 14 months of follow-up. During reoperation, dehiscence was found in the leaflet plication suture with moderate to important thickening at the leaflet edges. The reoperation consisted of a Ross procedure, with an uneventful recovery.

**Fig. 4:**
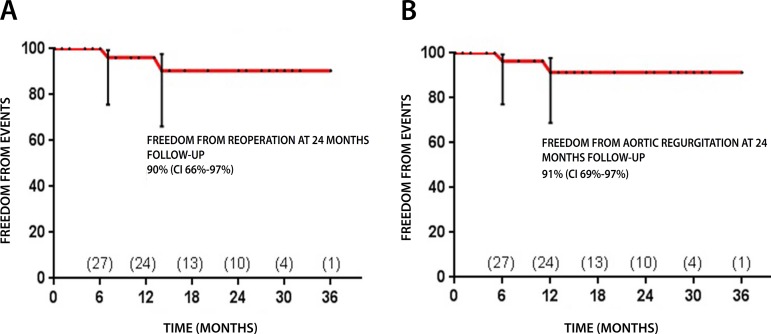
A - Freedom from reoperation. B - Freedom from aortic regurgitation.

By the Kaplan-Meier curve, 90% (CI 95% - 66%-97%) of patients are free from reoperation at 3 years of follow-up (Figure 4A).

### Functional Evaluation of the Repair

All patients underwent at least one echocardiogram in the late period, and the average echocardiographic follow-up was 10±6 months (min=0.5, max=29). Aside from the two patients who developed moderate or severe AR and were reoperated, all the others are stable with no, trivial or mild AI. During this short period of follow-up, cusp mobility is well preserved without signs of leaflet thickening. Leaflet coaptation has been maintained with effective coaptation height > 5 mm in all patients. The mean gradient in the late postoperative period was 11±9 mmHg (min=3, max=26), with no patient with a peak gradient greater than 40 mmHg. By the Kaplan-Meier curve, 91% of the patients (CI 95% - 69%-97%) are with normal functioning aortic valve two years after the procedure (Figure 4B).

## DISCUSSION

After four decades of accumulated experience, mitral valve repair is considered the procedure of choice in the surgical treatment of organic mitral regurgitation. However, its acceptance and applicability could only be widely generalized after the techniques have been standardized to allow consistent and reproducible early and late results^[15-21]^. Based on these concepts, and with better anatomical and physiological knowledge of the "aortic valve apparatus", several centers started using aortic valve repair with progressively more satisfactory results^[6,12,16,17]^. In our country, the only meaningful series of aortic valve sparing/repair operations was reported by Dias et al.^[22]^.

More recent studies have clarified in detail the anatomical relationships of the different structures of "aortic valve apparatus" and allowed the understanding of complex functional interrelationship between the valve and the aortic root. This has enabled the development of techniques that restore valve competence and allows physiological flow patterns in the left ventricular outflow tract and the aortic root^[8,18]^.

This study confirmed that aortic valve repair and/or aortic root replacement surgery sparing the native valve could be used with good immediate and short-term results in the surgical treatment of AI due to various etiologies. Early mortality reported by other centers ranged from 0.8% to 4.6%, but the comparison of results is quite difficult, given the differences in indications and clinical profile of the operated patients^[11,19-22]^. Our immediate mortality of 2.6% can be considered quite acceptable, given the complexity of some cases involving acute aortic dissection, reoperations and aneurysms involving the aortic arch. Our only death occurred in a patient with aneurysmal dilatation of the aortic root after previous correction of acute dissection, with important anatomical distortions and tissue fragility.

Restore valve competence systematically still remains a challenge, especially when accumulated surgical experience is still small^[6,23]^. Analysis of the data reported in the literature clearly show that the success of the operation depends upon the individual expertise and the volume of cases operated. Data from the Society of Thoracic Surgeons revealed that less than 5% of the analyzed centers performed more than 16 cases operations involving the aortic root annually, and that conservative operations represent less than 20% of the operated cases^[24]^. These numbers suggest that aortic valve repair should be concentrated in specialized reference centers dedicated in performing these techniques in a routine basis.

The incidence of intraoperative residual or recurrent AI with up to 1 year of follow-up can reach up to 30%, depending on the techniques employed^[6,23]^. Its most frequent causes are the residual prolapse of one or more cusps or failure to properly correct annular dilation over 25 mm^[5,6,19,25]^. In this sense, it is mandatory to have intraoperative monitoring with TEE, as the simple visual inspection or testing aortic valve with saline injection in its flaccid state often underestimate residual prolapses and are unsuitable for the determination of the ELH and the coaptation surface after procedure.

To achieve lasting results, it is necessary that at the end of the repair procedure not only the valve is competent, but also that the valve cusps coaptation line is in a plane corresponding to approximatelly half the height of the SV^[9,26]^. This concept was first described subjectively by El Khoury et al.^[9]^, and later reemphasized and objectively measurable with the concept of ELH introduced by Schaefers et al.^[26]^. This height can be measured during the operation with a specific caliper, and then confirmed by TEE on the beating heart after termination of CPB. With this methodology, we found the need to act aggressively to correct the FML of the leaflets in 59% of the cases, which certainly contributed to our low incidence of immediate postoperative AI, and maintenance of these results by up to 3 times years of follow-up.

In cases with marked dilation of the aortic root, there are controversies about the best technique to be employed, reimplantation *versus* remodeling^[13,14]^. The reimplantation technique has the advantages of always correcting annular dilatation and be more hemostatic, but it is more laborious and at the end of the procedure, the valve is inserted into a straight tube without SV that prevents the physiological movement of subcommissural triangles. In theory, these geometric changes would increase stress on the valve due to abnormal opening and closing movements of the leaflets, besides allowing direct contact of the cusps to the graft wall^[6,10]^. Despite these considerations, David^[16]^ demonstrated excellent long-term results with this technique, with 94% of patients free from reoperation and 74% free from moderate or severe AI after 20 years of follow-up. In addition, there is the possibility of creating neo SVs with some technical refinements such as in the David V modification or by the use of Dacron grafts that already have performed SV (Valsalva graft)^[27]^. On the other hand, the remodeling technique is faster, and is considered more physiological for creating new SV, avoiding some of the disadvantages of the reimplantation technique. However, this technique is more prone to bleeding, and does not correct any eventual associated annular dilatation^[9,17]^. Alternatively, as proposed by Lansac et al.^[6]^, the remodeling technique may be combined with a separate external annular support ring to correct the annular dimensions, a unique strategy to have the advantages of the two previous techniques simultaneously.

The judgment in which patients would be better served by conservative techniques is still rather speculative, since there are no randomized studies comparing the long-term results of valve repair against valve replacement with biological and/or mechanical prostheses^[1,3,22]^. However, some series with longterm follow-up seems to indicate that patients undergoing aortic valve repair have long-term survival approaching the normal matched population for age and gender, as opposed to conventional valve prostheses where life expectancy is reduced, especially in young patients^[4,5,16,25]^. In our series we had only one late death from cardiac causes, however, we will need larger series with longer follow-up to confirm these trend.

Our clinical observations also confirm the excellent quality of life after aortic valve repair. We observed excellent functional capacity, absence of thromboembolic, hemorrhagic and infectious complications, and a significant number of patients do not require any cardiotonic and/or anticoagulant medications.

One concern with aortic valve repair is the possible need for reoperation^[6,10]^. In our series, we had two reoperations for primary structural dysfunction that can be attributed to technical failures that could have been avoided if our experience with these procedures were greater. As a result of certain subjectivity and the somewhat "artistic" aspect of these operations, an initial learning curve could already be anticipated^[23]^. At any rate, our 90% freedom from reoperation at 24 months, compares favorably with other published studies^[6,23]^.

Aside from these two cases and despite the clinical and echocardiographic follow-up time is still very limited and do not allow more definitive conclusions, we have not observed increased levels of AI in the first 12-24 months of follow-up. Some demographic, anatomical and technical aspects such as age, diameter of the valve annulus, ELH post correction, commissural orientation lesser than 160º in bicuspid valves and the need to use patches to fix defects in the leaflets were described as risk factors for late AI and need for reoperation^[25]^. As our series is still early and has a small number of cases, we were unable to analyze the importance of these variables.

As noted in this study, 40% of patients were aged between 20-40 years. In our service, young aortic patients were routinely treated by the Ross operation. However, the analysis of our longterm results of up to 18 years of follow-up with this operation demonstrated unequivocally that patients with AI and annular dilatation had a greater need for reoperation due to recurrent AI or aneurysmal dilatation of the pulmonary autograft^[28]^. It seems to us that it is precisely in this sub-group of patients, the valve repair techniques may result in less need of late reoperations, but only with the continued clinical observation is that we will have more conclusive answers.

## CONCLUSION

In conclusion, the results presented here demonstrate that, despite representing an initial series, aortic valve repair and/ or aortic root replacement operations preserving the native valve can be performed safely and with satisfactory immediate and short-term results. A detailed anatomical knowledge and adherence to the technical principles of correction, always confirmed by intraoperative TEE seem essential to obtain consistent results. Continuous surveillance of patient outcomes and echocardiographic results will determine the role of these operations in patients with aortic insufficiency.

**Table:** 

Authors' roles & responsibilities	
FDAC	Analysis and/or data interpretation; statistical analysis; final approval of the manuscript
DFFC	Analysis and/or data interpretation; statistical analysis; final approval of the manuscript
ACBAC	Conception and design study; analysis and/or data interpretation; final approval of the manuscript
EMBF	Conception and design study; realization of operations and/or trials; analysis and/or data interpretation; final approval of the manuscript
VNC	Conception and design study; analysis and/or data interpretation; statistical analysis; final approval of the manuscript
SAVL	Conception and design study; analysis and/or data interpretation; final approval of the manuscript
ADAF	Conception and design study; analysis and/or data interpretation; statistical analysis; final approval of the manuscript
CC	Conception and design study; analysis and/or data interpretation; statistical analysis; final approval of the manuscript

## References

[1] Nishida T, Sonoda H, Oishi Y, Ushijima T, Tanoue Y, Nakashima A (2015). More than 20-year experience of Bentall operation with mechanical
prostheses for chronic aortic root aneurysm. Gen Thorac Cardiovasc Surg.

[2] Sioris T, David TE, Ivanov J, Armstrong S, Feindel CM (2004). Clinical outcomes after separate and composite replacement of the
aortic valve and ascending aorta. J Thorac Cardiovasc Surg.

[3] Etz CD, Girrbach FF, von Aspern K, Battellini R, Dohmen P, Hoyer A (2013). Longevity after aortic root replacement: is the mechanically
valved conduit really the gold standard for
quinquagenarians?. Circulation.

[4] Puvimanasinghe JP, Takkenberg JJ, Edwards MB, Eijkemans MJ, Steyerberg EW, Van Herwerden LA (2004). Comparison of outcomes after aortic valve replacement with a
mechanical valve or a bioprosthesis using microsimulation. Heart.

[5] Schäfers HJ (2015). Aortic valve repair: easy and reproducible?. J Thorac Cardiovasc Surg.

[6] Lansac E, Di Centa I, Sleilaty G, Crozat EA, Bouchot O, Hacini R (2010). An aortic ring: from physiologic reconstruction of the root to a
standardized approach for aortic valve repair. J Thorac Cardiovasc Surg.

[7] Kunzelman KS, Grande KJ, David TE, Cochran RP, Verrier ED (1994). Aortic root and valve relationships. Impact on surgical
repair. J Thorac Cardiovasc Surg.

[8] Labrosse MR, Beller CJ, Robicsek F, Thubrikar MJ (2006). Geometric modeling of functional trileaflet aortic valves:
development and clinical applications. J Biomech.

[9] El Khoury G, de Kerchove L (2013). Principles of aortic valve repair. J Thorac Cardiovasc Surg.

[10] David TE, Feindel CM, David CM, Manlhiot C (2014). A quarter of a century of experience with aortic valve-sparing
operations. J Thorac Cardiovasc Surg.

[11] Schäfers HJ, Aicher D, Langer F, Lausberg HF (2007). Preservation of the bicuspid aortic valve. Ann Thorac Surg.

[12] David TE (2015). Aortic valve repair and aortic valve-sparing
operations. J Thorac Cardiovasc Surg.

[13] David TE, Feindel CM (1992). An aortic valve-sparing operation for patients with aortic
incompetence and aneurysm of the ascending aorta. J Thorac Cardiovasc Surg.

[14] Sarsam MA, Yacoub M (1993). Remodeling of the aortic valve anulus. J Thorac Cardiovasc Surg.

[15] Adams DH, Anyanwu AC (2008). Seeking a higher standard for degenerative mitral valve repair:
begin with etiology. J Thorac Cardiovasc Surg.

[16] David TE (2013). Aortic valve sparing operations: outcomes at 20
years. Ann Cardiothorac Surg.

[17] Price J, De Kerchove L, Glineur D, Vanoverschelde JL, Noirhomme P, El Khoury G (2013). Risk of valve-related events after aortic valve
repair. Ann Thorac Surg.

[18] de Kerchove L, Jashari R, Boodhwani M, Duy KT, Lengelé B, Gianello P (2015). Surgical anatomy of the aortic root: Implication for
valve-sparing reimplantation and aortic valve annuloplasty. J Thorac Cardiovasc Surg.

[19] David TE, Armstrong S, Manlhiot C, McCrindle BW, Feindel CM (2013). Longterm results of aortic root repair using the reimplantation
technique. J Thorac Cardiovasc Surg.

[20] Yacoub MH, Gehle P, Chandrasekaran V, Birks EJ, Child A, Radley-Smith R (1998). Late results of a valve-preserving operation in patients with
aneurysms of the ascending aorta and root. J Thorac Cardiovasc Surg.

[21] De Paulis R, Scaffa R, Nardella S, Maselli D, Weltert L, Bertoldo F (2010). Use of the Valsalva graft and long-term follow-up. J Thorac Cardiovasc Surg.

[22] Dias RR, Mejia OA, Fiorelli AI, Pomerantzeff PM, Dias AR, Mady C (2010). Analysis of aortic root surgery with composite mechanical aortic
valve conduit and valve-sparing reconstruction. Rev Bras Cir Cardiovasc.

[23] Luciani GB, Casali G, Tomezzoli A, Mazzucco A (1999). Recurrence of aortic insufficiency after aortic root remodeling
with valve preservation. Ann Thorac Surg.

[24] Stamou SC, Williams ML, Gunn TM, Hagberg RC, Lobdell KW, Kouchoukos NT (2015). Aortic root surgery in the United States: a report from the
Society of Thoracic Surgeons database. J Thorac Cardiovasc Surg.

[25] Aicher D, Kunihara T, Abou Issa O, Brittner B, Gräber S, Schäfers HJ (2011). Valve configuration determines long-term results after repair of
the bicuspid aortic valve. Circulation.

[26] Schäfers HJ, Bierbach B, Aicher D (2006). A new approach to the assessment of aortic cusp
geometry. J Thorac Cardiovasc Surg.

[27] Demers P, Miller DC (2004). Simple modification of "T. David-V" valve-sparing aortic root
replacement to create graft pseudosinuses. Ann Thorac Surg.

[28] da Costa FD, Takkenberg JJ, Fornazari D, Balbi Filho EM, Colatusso C, Mokhles MM (2014). Long-term results of the Ross operation: an 18-year single
institutional experience. Eur J Cardiothorac Surg.

